# Impact of prehospital stroke triage implementation on patients with intracerebral hemorrhage

**DOI:** 10.1177/17562864231168278

**Published:** 2023-05-10

**Authors:** Tove Almqvist, Anna Falk Delgado, Christina Sjöstrand, Niaz Ahmed, Annika Berglund, Einar Eriksson, Michael V. Mazya

**Affiliations:** Department of Clinical Neuroscience, Karolinska Institutet, Karolinska University Hospital, 171 64 Stockholm, Sweden; Department of Neurology, Karolinska University Hospital, Stockholm, Sweden; Department of Neurology, Danderyd Hospital, Stockholm, Sweden; Department of Clinical Neuroscience, Karolinska Institutet, Karolinska University Hospital, Stockholm, Sweden; Department of Neuroradiology, Karolinska University Hospital, University Hospital; Department of Clinical Neuroscience, Karolinska Institutet, Karolinska University Hospital, Stockholm, Sweden; Department of Neurology, Karolinska University Hospital, Stockholm, Sweden; Department of Neurology, Danderyd Hospital, Stockholm, Sweden; Department of Clinical Neuroscience, Karolinska Institutet, Karolinska University Hospital, Stockholm, Sweden; Department of Neurology, Karolinska University Hospital, Stockholm, Sweden; Department of Clinical Neuroscience, Karolinska Institutet, Karolinska University Hospital, Stockholm, Sweden; Department of Neurology, Karolinska University Hospital, Stockholm, Sweden; Department of Clinical Neuroscience, Karolinska Institutet, Karolinska University Hospital, Stockholm, Sweden; Department of Neurology, Karolinska University Hospital, Stockholm, Sweden; Department of Clinical Neuroscience, Karolinska Institutet, Karolinska University Hospital, Stockholm, Sweden; Department of Neurology, Karolinska University Hospital, Stockholm, Sweden

**Keywords:** acute stroke, intracerebral hemorrhage, neurosurgery, prehospital emergency care, triage

## Abstract

**Background:**

Little is known about how prehospital triage using large vessel occlusion (LVO) stroke prediction scales affects patients with intracerebral hemorrhage (ICH).

**Objectives:**

We aimed to investigate whether the Stockholm Stroke Triage System (SSTS) implemented in 2017 has affected timing and outcomes of acute ICH neurosurgery, and to assess system triage accuracy for ICH with a neurosurgical indication or LVO thrombectomy.

**Design:**

Observational cohort study.

**Methods:**

In the Stockholm Region, we compared surgical timing, functional outcome, and death at 3 months in patients transported by code-stroke ground ambulance who had ICH neurosurgery, 2 years before *versus* 2 years after SSTS implementation. We also calculated triage precision metrics for treatment with either ICH neurosurgery or thrombectomy.

**Results:**

A total of 36 patients undergoing ICH neurosurgery were included before SSTS implementation and 30 after. No significant difference was found in timing of neurosurgery [median 7.5 (4.9–20.7) *versus* 9.1 (6.1–12.5) h after onset], distribution of functional outcomes (median 4 *versus* 4), and death at 3 months [3/29 (9%) *versus* 5/35 (17%)] before *versus* after implementation, respectively. The SSTS routed a larger proportion of patients subsequently undergoing ICH neurosurgery directly to the comprehensive stroke center: 13/36 (36%) before *versus* 18/30 (60%) after implementation. Overall system triage accuracy for ICH neurosurgery or thrombectomy was high at 90%, with 92% specificity and 65% sensitivity.

**Conclusion:**

The SSTS, initially designed for prehospital LVO stroke triage, routed more patients with neurosurgical indication for ICH directly to the comprehensive stroke center. This did not significantly affect surgical timing or outcomes.

## Introduction

Recently updated intracerebral hemorrhage (ICH) guidelines from the American Heart Association/American Stroke Association recommend prehospital tools to recognize stroke and grade its severity.^1^ Meanwhile, studies of prehospital severity-based algorithms on ICH are lacking. This is highlighted in the guidelines, which emphasize the need for research on the impact of regionalized large vessel occlusion (LVO) stroke pathways on ICH patients.^1^ Comprehensive stroke centers (CSCs) receive a larger proportion of ICH patients after implementation of prehospital LVO protocols, owing to higher symptom severity in ICH compared with ischemic stroke and stroke mimics.^1–3^ Avoiding interhospital transfers in ICH has been reported to reduce the risk of deterioration during transport and decrease costs.^4–6^ It is yet unknown whether symptom-based prehospital triage of patients to a CSC leads to more rapid initiation of ICH treatments only available at CSCs, specifically acute neurosurgery.

In 2017, the Stockholm Region implemented the Stockholm Stroke Triage System (SSTS), aiming to identify patients with LVO stroke and transport them directly to the CSC, bypassing more proximal primary stroke centers (PSCs). The SSTS reduced time from onset to endovascular thrombectomy (EVT) by 69 min without delaying intravenous thrombolysis (IVT), and significantly improved outcomes in EVT.^7,8^ Of nearly 3000 patients annually taken to hospital by code-stroke ambulance in the Stockholm Region, 8% have previously been shown to suffer from ICH, 4% a subarachnoid or subdural hemorrhage, 44% an ischemic stroke or transient ischemic attack, and 44% a stroke mimic.^9^

First, we aimed to evaluate whether timing and outcome of acute ICH neurosurgery changed after SSTS implementation. Second, we aimed to expand previous results on SSTS accuracy for identification of patients needing EVT, by investigating the system’s accuracy for patients requiring either EVT or acute ICH neurosurgery, and assess differences between triage-positive and triage-negative ICH patients.

## Materials and methods

### Study groups and patient definitions

To calculate timing and outcomes of acute ICH neurosurgery before *versus* after SSTS implementation, we included cases operated 2 years before and 2 years after implementation of the SSTS: 10 October 2015–9 October 2017 *versus* 10 October 2017–9 October 2019. Inclusion criteria were symptom onset within the Stockholm Region and transport by code-stroke ground ambulance. Local guidelines for acute ICH neurosurgery remained unchanged at the CSC throughout the 4-year study period. Treatment decisions were made on a case-by-case basis. Surgical aims were to relieve life-threatening mass effect and/or elevated intracranial pressure. Cerebellar ICH could be eligible for surgery with hematoma diameter >3 cm, with reduced and deteriorating level of consciousness (LOC), hydrocephalus, or brainstem compression. Lobar ICH could be eligible for surgery in cases with reduced and deteriorating LOC due to mass effect. Intraventricular hemorrhage (IVH) could be surgically treated in the presence of clinical and radiological signs of disturbed cerebrospinal fluid circulation, in the absence of a large deep ICH. For patients with deep ICH, severe comorbidity or low pre-ICH level of function, coagulopathy caused by disease (e.g. leukemia or liver failure), or very low initial Glasgow Coma Scale (GCS) score, surgery was generally not recommended. Acute neurosurgery was defined as hematoma evacuation, external ventricular drainage (EVD), or insertion of an intracranial pressure monitor. We excluded ICH patients undergoing purely endovascular treatment to prevent hematoma recurrence, such as embolization of an arteriovenous malformation or fistula. The reason for this was that such treatments are generally performed within a time span where potential time gains from prehospital PSC bypass were deemed unlikely to have a clinically meaningful influence.^10,11^ For patients operated multiple times, the first surgery was used for time metric calculation.

Calculation of triage accuracy (based on specificity, sensitivity, and positive and negative predictive values) defined treatment positive status as either arterial puncture for LVO thrombectomy or acute neurosurgery for ICH. For this aim, we used the original SSTS study data set from the first year after system implementation, 10 October 2017–9 October 2018. In this period, 2909 patients were transported by code-stroke ground ambulance within the Stockholm Region. Of these, four opted out from study participation, leaving 2905 included cases in the data set for triage accuracy calculation.^7^ We deemed it futile to consider LVO thrombectomy as treatment negative and calculate triage accuracy for ICH neurosurgery alone, since only 8% of patients had an ICH.^9^

### Stockholm Stroke Triage System

**Figure 1.**
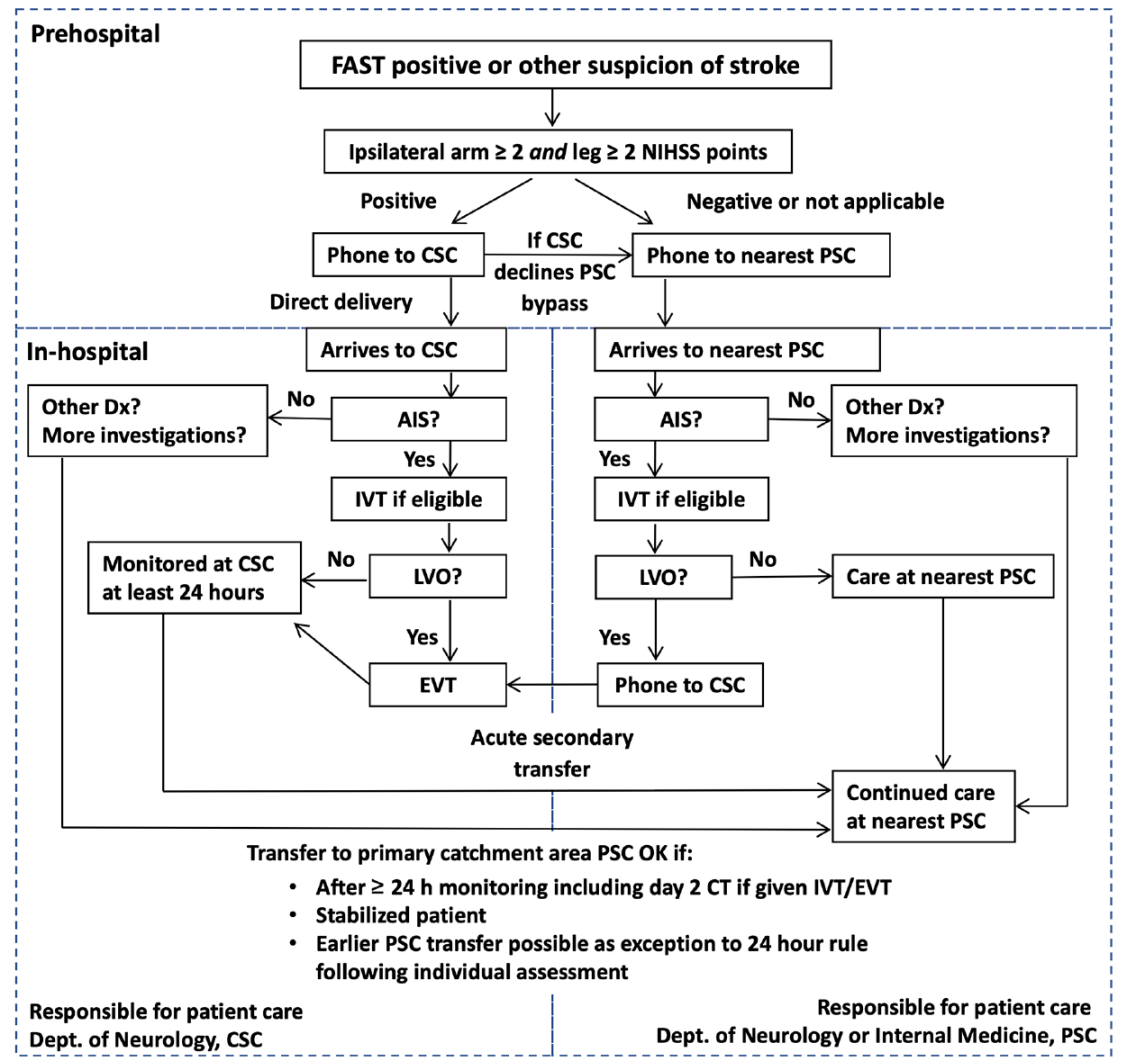
Flowchart of the SSTS. AIS, acute ischemic stroke; CSC, comprehensive stroke center; CT, computed tomography; EVT, endovascular thrombectomy; FAST, face-arm-speech-time; IVT, intravenous thrombolysis; LVO, large vessel occlusion; NIHSS, NIH Stroke Scale; PSC, primary stroke center; SSTS, Stockholm Stroke Triage System.

The SSTS is a three-step prehospital algorithm, visualized in Figure 1, previously published in detail.^7,12^ First, a suspicion of stroke is made by an ambulance nurse by using a Swedish version of the face-arm-speech-time (FAST) test. Step 2 is a test for moderate to severe hemiparesis.^13^ The ambulance nurse tests the patient for ⩾2 NIH Stroke Scale points each for an arm and the ipsilateral leg, called the Arm-2-Leg-2-test (A2L2). Step 3 is an ambulance to hospital teleconsultation, mandatory in all cases regardless of the result of the A2L2 test.

In A2L2-positive patients, ambulance staff consults the CSC stroke physician, who assesses whether a stroke suspicion is reasonable and checks the region-wide electronic health record system for contraindications to thrombectomy, for example, prestroke modified Rankin Scale (mRS) 4-5 or extensive comorbidity resulting in life expectancy less than 3 months. A positive A2L2 test, no EVT contraindications, and low probability of a stroke mimic make a patient triage-positive, bypassing the nearest PSC.

In A2L2-negative cases, the ambulance consults the stroke physician on call at the nearest PSC. This serves as a prenotification and A2L2-negative cases are routinely taken to the PSC. Unconscious patients, in whom the A2L2 test is inapplicable, are by default defined as triage-negative, as are patients requiring immediate resuscitation for unstable airway, breathing, or circulation. These cases are transported to the nearest PSC with physician prenotification. When the CSC was the most proximal hospital, both A2L2 negative and test inapplicable cases were teleconsulted and routed to the CSC. These were classified as triage-negative for research purposes.

Before the SSTS, patients with suspected stroke were taken to the most proximal hospital (PSC or CSC) regardless of symptom severity. After an initial diagnostic work-up and acute management, patients with indication for either EVT or ICH neurosurgery were transferred to the CSC.

### Data collection and statistics

Data were collected on demographics, medical history and pre-ICH medications, prehospital clinical parameters, in-hospital clinical and radiological parameters, treatments, time metrics, and in operated patients mRS scores at 3 months. Data sources included the local stroke quality registry using the Riksstroke platform, and prehospital and in-hospital electronic health records. The Karolinska Sectra Picture Archiving and Communication System was used for examining radiological scans. ICH volume was calculated semi-automatically from acute computed tomography scans, excluding IVH volume. For patients with pure IVH, no ICH volume was calculated.

The Mann–Whitney *U* test was used for comparing medians and interquartile range (IQR) for continuous and ordinal variables. Missing data were excluded. For categorical variables, the Pearson chi-square or two-sided Fisher’s exact test was used as appropriate. Univariate ordinal logistic regression was used to analyze differences in the distribution of mRS scores. We considered *p* values < 0.05 significant. Statistical analysis was performed using Rstudio.^14^ In sensitivity analyses, we assessed whether timing and outcomes of neurosurgery changed between patients admitted to a PSC before SSTS implementation and patients who bypassed a PSC after SSTS implementation.

## Results

**Figure 2.**
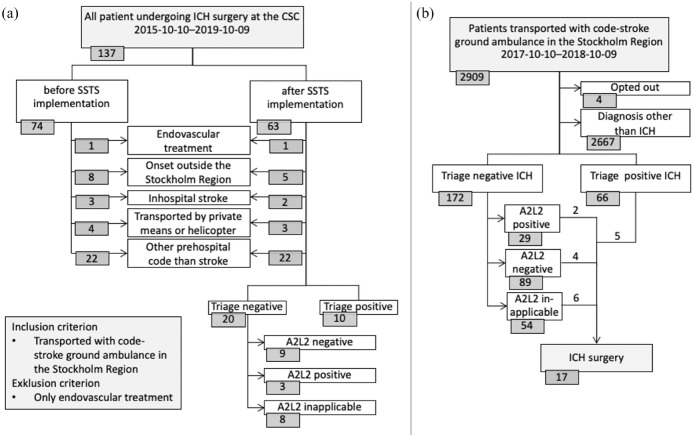
Flowchart of (a) patients undergoing ICH neurosurgery and (b) patients during the first year after SSTS implementation. Triage-positive defined as A2L2-positive and accepted for PSC bypass by CSC stroke physician. A2L2, Arm-2-Leg-2-test; CSC, comprehensive stroke center; ICH, intracerebral hemorrhage; PSC, primary stroke center; SSTS, Stockholm Stroke Triage System; TIA, transient ischemic attack.

The number of patients transported by code-stroke ambulance, undergoing acute ICH neurosurgery, was 36 during 2 years before SSTS implementation and 30 two years after. These cases made up 51% of all 129 patients undergoing ICH neurosurgery at the CSC during this period. The remaining 63 operated cases were excluded due to reasons such as not having a prehospital stroke suspicion or being transferred from another region (Figure 2(a)). During the 2 years before implementation, 74/272 (27%) of all patients with ICH at the CSC underwent neurosurgery, compared with 63/254 (25%) after implementation. Clinical characteristics (Table 1) were similar between the before and after SSTS groups, with median age 58 *versus* 63 years (*p* = 0.41), median initial in-hospital GCS 13 in both groups, and median initial ICH volume 34 *versus* 25 ml, *p* = 0.13. The hematoma location was infratentorial in 5/36 (14%) and 4/30 (13%), respectively, *p* = 1.0. IVH was present in 25/36 (69%) and 18/30 (60%) respectively, *p* = 0.42.

**Table 1.** Patients who underwent neurosurgery for ICH 2 years before *versus* 2 years after SSTS implementation.

	Before SSTS, *n* = 36		After SSTS, *n* = 30		*p*
	*n*	% or median (IQR)	*n*	% or median (IQR)	
Age	36/36	58 (46–66)	30/30	63 (49–71)	0.41
Sex (female)	14/36	39%	11/30	37%	0.85
Medical history					
Previous ICH	1/36	3%	1/30	3%	1
Previous ischemic stroke	3/36	8%	3/30	10%	1
Atrial fibrillation	4/36	11%	2/30	7%	0.68
Prestroke dependency	3/36	8%	1/30	3%	0.62
Pre-ICH medication					
Oral anticoagulants	2/36	6%	1/30	3%	1
Antiplatelets	6/36	17%	10/30	33%	0.12
Antihypertensive	19/36	53%	13/30	43%	0.44
A2L2 status					
Positive	–		13/30	43%	
Negative	–		9/30	30%	
Inapplicable	–		8/30	27%	
Triage-positive in the SSTS	–		10/30	33%	
Secondary transport to CSC	23/36	64%	12/30	40%	0.053
Known exact onset time	27/36	75%	17/30	57%	0.12
Symptoms on wake-up	3/36	8%	5/30	17%	0.46
NIHSS	9/36	19 (6–20)	19/30	12 (7–18)	0.46
GCS	36/36	13 (8–14)	30/30	13 (9–14)	0.61
Systolic blood pressure	35/36	200 (160–220)	30/30	191 (157–216)	0.51
ICH volume (ml)^a^	35/36	34 (18–75)	30/30	25 (13–49)	0.13
Supratentorial	30/36	39 (23–77)	26/30	28 (13–50)	0.07
Infratentorial	5/36	17 (4–19)	4/30	19 (14–24)	0.54
IVH	25/36	69%	18/30	60%	0.42
Hydrocephalus	20/36	56%	17/30	57%	0.93
Logistics (hours)					
Onset to surgery, incl. LKW	34/36	7.5 (4.9–20.7)	29/30	9.1 (6.1–12.5)	0.86
Onset to surgery, only known onset time	27/36	6.9 (4.7–20.3)	17/30	9 (5.1–11.2)	0.80
Door to surgery^b^	36/36	5.1 (3.3–13.6)	30/30	5.7 (3.6–10)	0.98
Type of surgery					
Hematoma evacuation^c^	20/36	56%	15/30	50%	0.76
EVD only	15/36	42%	14/30	47%	0.68
ICP monitor only	1/36	3%	0/30	0%	–

A2L2, Arm-2-Leg-2-test; CSC, comprehensive stroke center; EVD, external ventricular drainage; GCS, Glasgow Coma Scale; ICH, intracerebral hemorrhage; ICP: intracranial pressure; IQR: interquartile range; IVH, intraventricular hemorrhage; LKW, last-known-well; NIHSS, NIH Stroke Scale; SSTS, Stockholm Stroke Triage System.

a Patients with solitary IVH not included in calculations.

b Calculated from first hospital arrival.

c With or without EVD or ICP monitor.

**Figure 3.**
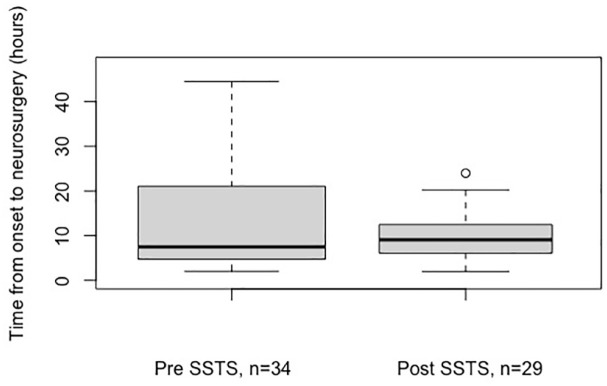
Median and interquartile range for timing of ICH neurosurgery in patients with ICH before and after SSTS implementation. Maximum values not shown. ICH, intracerebral hemorrhage; SSTS, Stockholm Stroke Triage System.

**Figure 4.**
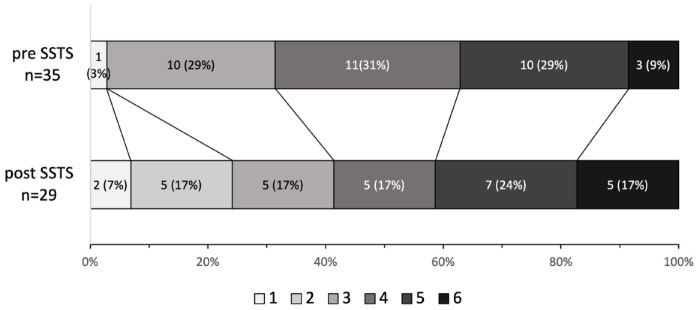
Modified Rankin Scale at 3 months for patients undergoing ICH neurosurgery before *versus* after SSTS implementation. ICH, intracerebral hemorrhage; SSTS, Stockholm Stroke Triage System.

**Figure 5.**
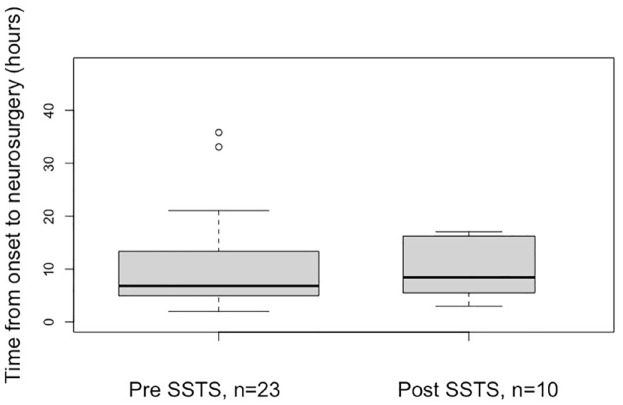
Median and interquartile range for timing of ICH neurosurgery comparing patient admitted to a PSC before SSTS implementation, and triage-positive patients who actively bypassed a PSC after SSTS implementation. ICH, intracerebral hemorrhage; PSC, primary stroke center; SSTS, Stockholm Stroke Triage System.

**Figure 6.**
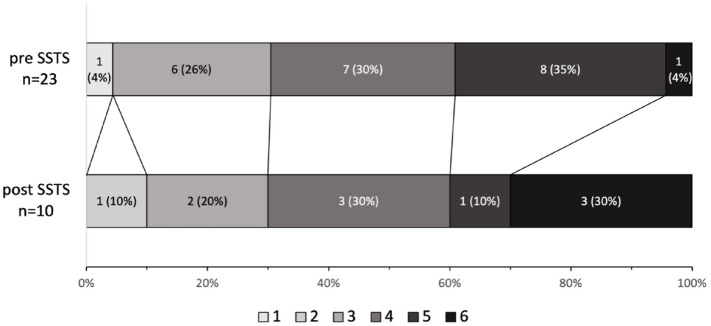
Modified Rankin Scale at 3 months for patients undergoing ICH neurosurgery comparing patient admitted to a PSC before SSTS implementation, and triage-positive patients who actively bypassed a PSC after SSTS implementation. ICH, intracerebral hemorrhage; PSC, primary stroke center; SSTS, Stockholm Stroke Triage System.

Before SSTS, 23/36 (64%) patients underwent secondary transportation to the CSC from a PSC, compared with 12/30 (40%) after, *p* = 0.053. Median time from first hospital arrival to acute neurosurgery was 5.1 (IQR = 3.3–13.6) *versus* 5.7 (IQR = 3.6–10) h, before *versus* after SSTS, *p* = 0.98 (Figure 3). Mortality at 3 months was 3/35 (9%) *versus* 5/29 (17%) in the pre- and post-SSTS group, respectively, *p* = 0.45. The overall distribution across the full range of mRS scores did not differ significantly (Figure 4), *p* = 0.64. One patient in each group was lost to follow-up due to living abroad. After comparing surgical timing and outcomes of patients admitted to a PSC before SSTS (23/36, 64%) to patients who were triage-positive and actively bypassed a PSC (10/30, 33%), our results did not change (Figures 5 and 6).

Of 2905 prehospital code-stroke patients included in the first SSTS year, 66 triage-positive and 172 triage-negative patients received an ICH diagnosis (Figure 2(b)). Triage-positive ICH patients were younger (median 72.5 *versus* 77, *p* = 0.01), had a higher median NIH Stroke Scale (NIHSS) score (15 *versus* 9, *p* < 0.001), and less commonly prestroke dementia or cognitive impairment (5% *versus* 19%, *p* = 0.04). Other characteristics did not differ significantly, except differences explained by group definitions, such as A2L2 status (Table 2). ICH neurosurgery was similarly frequent between triage-positive and triage-negative patients, 5/66 (8%) and 12/172 (7%). Of 12 triage-negative patients who underwent neurosurgery, 4/12 (33%) had a GCS ⩽ 8 on first hospital arrival. Triage accuracy for CSC-specific treatment defined as thrombectomy or ICH neurosurgery was 90.4%, with a high specificity (91.6%) and moderate sensitivity (65.4%). This can be compared with previously published accuracy of 90.6% (91.6% specificity and 70.6% sensitivity) for EVT alone.^7^ Sensitivity, specificity, and positive and negative predictive values are shown in Table 3.

**Table 2.** Patients with ICH within the SSTS.

	Triage-positive ICH, *n* = 66		Triage-negative ICH, *n* = 172		*p*
	*n*	% or median (IQR)	*n*	% or median (IQR)	
Age	66/66	72.5 (65–79)	172/172	77 (68–83)	0.01
Sex (female)	25/66	38%	86/172	50%	0.09
Medical history					
Previous ICH	2/66	3%	13/172	8%	0.25
Previous ischemic stroke	9/66	14%	29/172	17%	0.54
Previous high blood pressure	49/66	74%	123/172	72%	0.67
Atrial fibrillation	18/66	27%	33/172	19%	0.17
Dementia or cognitive impairment	3/66	5%	33/172	19%	0.04
Pre-ICH medication					
Oral anticoagulants	16/66	24%	35/172	20%	0.51
Antiplatelets	16/66	24%	47/172	27%	0.63
Antihypertensive	41/66	65%	108/172	63%	0.92
A2L2 test					
Positive	66/66	100%	29/172	17%	<0.001
Negative	0/66	0%	89/172	52%	<0.001
Not applicable	0/66	0%	54/172	31%	<0.001
First hospital: CSC	66/66	100%	27/172	16%	<0.001
Secondary transport	–		12/172	7%	–
Known exact onset time	39/66	59%	84/172	49%	0.16
NIHSS	60/66	15 (11–19)	138/172	9 (4–16)	<0.001
GCS	66/66	14 (12–15)	172/172	13 (8–15)	0.06
Systolic blood pressure	61/66	175 (159–195)	172/172	170 (150–200)	0.45
Neurosurgery	5/66	8%	12/172	7%	1
Onset to surgery (hours)	5/66	16.3 (7.1–17.1)	12/172	10.1 (5.7–17.6)	0.71
Door to surgery^a^ (hours)	5/66	5 (2.5–6)	12/172	4.6 (3.5–11)	0.96

A2L2: Arm-2-Leg-2-test; CSC: comprehensive stroke center; GCS: Glasgow Coma Scale; ICH: intracranial hemorrhage; NIHSS: NIH Stroke Scale; SSTS: Stockholm Stroke Triage System.

a Calculated from first hospital arrival.

**Table 3.** Triage accuracy.

	Thrombectomy or neurosurgery		Thrombectomy	
	%	95% CI	%	95% CI
Sensitivity	65.4	56.8–73.4	70.6	61.5–78.6
Specificity	91.6	90.5–92.6	91.4	90.3–92.4
Positive predictive value	27.7	24.4–31.3	26.0	22.9–29.4
Negative predictive value	98.2	97.7–98.6	98.6	98.2–99.0
Accuracy	90.4	89.3–91.4	90.6	89.5–91.6

CI, confidence interval.

Among 29 A2L2-positive ICH patients not undergoing PCS bypass, median age was 84 years, 11 (38%) had known onset time, and 9 (31%) had dementia or cognitive impairment. In 10 (34%) of these 29 cases, there was a protocol violation in the form of no teleconsultation with the CSC stroke physician, including one patient who subsequently had ICH neurosurgery after secondary transport. One triage-negative but A2L2-positive patient who had ICH neurosurgery was initially declined Primary Stroke Center (PSC) bypass due to no available bed at the CSC.

## Discussion

We found no change in timing or outcome of ICH neurosurgery after implementation of region-wide stroke triage for PSC bypass. This was despite more ICH patients being routed directly to the CSC, bypassing the nearest PSC. The optimal timing of neurosurgery in ICH is still not established.^1^ In the STICH I and II trials, 60% of patients underwent surgery after 24 h and patients had the highest probability of mRS 0-3 when operated 62 h after onset.^15,16^ In relation to these results, the median surgical timing (7.5 and 9.1 h) in our study was quite early. Meanwhile, in a meta-analysis of surgical intervention in patients with cerebellar ICH, median time from onset to surgery was 9–9.5 h.^17^ Luzzi *et al.* analyzed both cerebellar and supratentorial ICH and suggested the optimal timing for surgery to be within 7–24 h, based on higher rates of complications in earlier and later time windows.^18^ It has been proposed that the group of patients who ultimately undergo neurosurgery is too heterogeneous to uniformly recommend one type of surgery and timing over another.^19^

Regarding the SSTS, we found that overall triage accuracy was largely unchanged and remained high when ICH neurosurgery was combined with LVO stroke thrombectomy as the outcome of interest. We were unable to find previous publications evaluating this topic. Several studies of in-hospital ICH patient cohorts have looked for predictors of neurosurgery or neurointensive care. Some use subitems of the ICH Score, which rely on information obtained after acute imaging.^20,21^ Patel *et al.*^20^ additionally described prior antiplatelet treatment as a risk factor for needing surgery. To utilize CSC and prehospital resources efficiently, it is important to identify patients in need of CSC care as early as possible and avoid unnecessary interhospital transfer.^5^ Kaleem *et al.* proposed that patients with a supratentorial ICH volume < 15 ml, no IVH, and GCS > 13 have a low likelihood of needing surgery or neurointensive care, and thus can safely avoid transfer to the CSC.^4^ Our results showed that the SSTS routs the majority of ICH patients without indication for surgery correctly to the PSC (160/221, 72%).

We have found no previous studies investigating the use of prehospital LVO scales to identify patients with ICH needing CSC-specific care such as neurosurgery. Initial studies of Rapid Arterial Occlusion Evaluation (RACE) and the Ambulance Clinical Triage for Acute Stroke Treatment (ACT-FAST) algorithms saw a larger proportion of ICH patients routed to the CSC, but these studies did not differentiate between ICH with different needs of care.^2,3^ Mobile stroke units can shorten time to ICH diagnosis and provide details of hemorrhage location and presence of IVH before arriving at a hospital, likely reducing interhospital transfers.^22^ In the future, serum biomarkers could be used for prehospital identification of ICH without mobile stroke units, but the need for identification of patients with surgical indication remains.^23^

As a consequence of the SSTS algorithm, triage-positive ICH patients had higher NIHSS scores than triage-negative ICH, in line with previous findings in the entire code-stroke SSTS cohort.^13^ Meanwhile, the A2L2 test is inapplicable in unconscious patients and those with bilateral paresis, who are treated as triage-negative in the prehospital phase. In the SSTS workflow, such cases are taken to the nearest PSC for assessment of an indication for intubation and initial diagnostic work-up, both in order to avoid delays to resuscitation, and because unconsciousness is caused by stroke in only a minority of patients.^24^ We have previously shown that these cases rarely (in 1%) have an indication for thrombectomy.^7^ In this study, the A2L2 test was inapplicable in 31% of triage-negative ICH cases routed to a PSC. This is reflected by the finding that one in four triage-negative cases had a GCS score < 8 (median = 13, IQR = 8–15). Despite that GCS 5-12 has been reported as predictive for neurosurgery, triage-negative patients did not have surgery to a greater extent.^1,18^

As for study limitations, we found that prehospital recognition of stroke was moderate for patients with ICH in need of neurosurgery. In 44/110 (40%) surgically treated ICH cases transported by ambulance, there was no documented prehospital suspicion of stroke. In these cases, the most common prehospital transportation codes were reduced level of consciousness, vertigo, and seizure. Similarly, Kleindorfer *et al.*^25^ showed that among patients with ICH or subarachnoid hemorrhage, 69% are FAST positive, compared with 91% for ischemic stroke. According to Oostema *et al.*,^26^ 65% of ICH patients were recognized by ambulance staff when mainly using the Cincinnati Prehospital Stroke Scale. Compared with other prehospital scales aimed at recognizing LVO stroke, the SSTS provides a higher specificity and a lower sensitivity.^27^ This is explained by the perceived need at the design phase, for a high negative predictive value system to avoid overwhelming CSC capacity. This was achieved by requiring a severe symptom (hemiparalysis) and a teleconsultation, which resulted in a relatively low proportion (11%) of ambulance code-stroke patients being triage-positive. This can be compared with RACE scale scores ⩾ 5, a commonly used triage-positive cutoff that classifies 48% of cases as triage-positive.^28^ What constitutes the optimal relationship between sensitivity and specificity may depend on specific regional and organizational circumstances.^29^ As the SSTS did not introduce any changes in stroke recognition tools or in-hospital practices, timing and outcomes of neurosurgery should not be affected for patients who did not go through a ground ambulance code-stroke pathway. This was, however, not investigated. In line with this, we only evaluated outcomes in patients with surgically treated ICH, since all hospitals in the region provide intensive and stroke unit care also for ICH patients, with unchanged guidelines throughout the duration of the study. Furthermore, triage status in the SSTS is affected by pre-stroke functional impairment or severe comorbidity. Accordingly, we found that triage-negative patients had a pre-stroke diagnosis of dementia or cognitive impairment to a greater extent. We, however, do not know the exact cause of bypass decline for all A2L2-positive patients. In the 10 cases of protocol violation, we have found no exact cause as to why the ambulance staff did not call the CSC physician. It is possible that it is partly due to failure to reach all ambulance staff with information and training regarding the triage system, with the number of ambulance clinicians being approximately 700 during the study period. A limitation in generalizability is that our study was conducted within a specific regional system of care, and the results may be inapplicable in areas with different geographical and organizational circumstances. Indications for neurosurgery may differ between regions and countries, and change over time. Moreover, low sample size may have reduced our likelihood of detecting significant differences between groups.

## Conclusion

The Stockholm Stroke Triage System was not associated with a significant change in timing or outcome of neurosurgical treatment in patients with ICH, despite more patients being routed directly to the CSC. The SSTS has a high accuracy for patients with indication for thrombectomy or ICH neurosurgery at the CSC, based on high specificity and moderate sensitivity. We encourage researchers from other regions with prehospital LVO stroke triage to evaluate the impact of their triage systems on ICH care.

## Declarations

**Ethics approval and consent to participate:** Swedish Ethical Review Authority approval was obtained (dnr 2021-06163-01 and 2917/374-31/2) and the need for consent was waived due to the retrospective nature of the study.

**Consent for publication:** Not applicable.

**Availability of data and materials:** Anonymized data can be made available upon reasonable request from a qualified researcher.

## References

[1] Greenberg SM Ziai WC Cordonnier C , et al. 2022 Guideline for the management of patients with spontaneous intracerebral hemorrhage: a guideline from the American Heart Association/American Stroke Association. Stroke 2022; 53(7): e282–e361. 10.1161/STR.0000000000000407 35579034

[2] Pérez de la Ossa N Carrera D Gorchs M , et al. Design and validation of a prehospital stroke scale to predict large arterial occlusion: the rapid arterial occlusion evaluation scale. Stroke 2014; 45: 87–91. 24281224 10.1161/STROKEAHA.113.003071

[3] Zhao H Pesavento L Coote S , et al. Ambulance clinical triage for acute stroke treatment. Stroke 2018; 49: 945–951. 29540611 10.1161/STROKEAHA.117.019307

[4] Kaleem S Lutz MW Hernandez CE , et al. A triage model for interhospital transfers of low risk intracerebral hemorrhage patients. J Stroke Cerebrovasc Dis 2021; 30: 105616. 33476961 10.1016/j.jstrokecerebrovasdis.2021.105616

[5] Nakagawa K Galati A Juarez DT. The excess cost of interisland transfer of intracerebral hemorrhage patients. Am J Emerg Med 2015; 33: 512–515. 25624077 10.1016/j.ajem.2015.01.001 PMC4409479

[6] Fan JS Chen YC Huang HH , et al. Interhospital transfer neurological deterioration in patients with spontaneous intracerebral haemorrhage: incidence and risk factors. Postgrad Med J 2017; 93: 349–353. 27733674 10.1136/postgradmedj-2016-134463

[7] Mazya MV Berglund A Ahmed N , et al. Implementation of a prehospital stroke triage system using symptom severity and teleconsultation in the Stockholm Stroke Triage Study. JAMA Neurol 2020; 77: 691–699. 32250423 10.1001/jamaneurol.2020.0319 PMC7136864

[8] Keselman B Berglund A Ahmed N , et al. The Stockholm Stroke Triage Project: outcomes of endovascular thrombectomy before and after triage implementation. Stroke 2022; 53: 473–481. 34583527 10.1161/STROKEAHA.121.034195

[9] Sjöö M Berglund A Sjöstrand C , et al. Prehospital stroke mimics in the Stockholm Stroke Triage System. Front Neurol 2022; 13: 939618. 36062015 10.3389/fneur.2022.939618 PMC9433744

[10] Jafar JJ Rezai AR. Acute surgical management of intracranial arteriovenous malformations. Neurosurgery 1994; 34: 8–12; discussion 12–13. 8121572

[11] Pavesi G Rustemi O Berlucchi S , et al. Acute surgical removal of low-grade (Spetzler-Martin I-II) bleeding arteriovenous malformations. Surg Neurol 2009; 72: 662–667. 19604554 10.1016/j.surneu.2009.03.035

[12] Almqvist T Berglund A Sjöstrand C , et al. Prehospital triage accuracy in patients with stroke symptoms assessed within 6 to 24 hours or with an unknown time of onset. Stroke 2021; 52: 1441–1445. 33641383 10.1161/STROKEAHA.120.033676

[13] Cooray C Mazya MV Bottai M , et al. Are you suffering from a large arterial occlusion? Please raise your arm! Stroke Vasc Neurol 2018; 3: 215–221. 30637127 10.1136/svn-2018-000165 PMC6312073

[14] R Core Team. R: a language and environment for statistical computing. Vienna: R Foundation for Statistical Computing, 2022. https://www.R-project.org/

[15] Sondag L Schreuder FHBM Boogaarts HD , et al. Neurosurgical intervention for supratentorial intracerebral hemorrhage. Ann Neurol 2020; 88: 239–250. 32239722 10.1002/ana.25732 PMC7497162

[16] Polster SP Carrión-Penagos J Lyne SB , et al. Intracerebral hemorrhage volume reduction and timing of intervention versus functional benefit and survival in the MISTIE III and STICH trials. Neurosurgery 2021; 88: 961–970. 33475732 10.1093/neuros/nyaa572 PMC8190461

[17] Kuramatsu JB Biffi A Gerner ST , et al. Association of surgical hematoma evacuation vs conservative treatment with functional outcome in patients with cerebellar intracerebral hemorrhage. JAMA 2019; 322: 1392–1403. 31593272 10.1001/jama.2019.13014 PMC6784768

[18] Luzzi S Elia A Del Maestro M , et al. Indication, Timing, and surgical treatment of spontaneous intracerebral hemorrhage: systematic review and proposal of a management algorithm. World Neurosurg 2019; 124: e769–e778. 10.1016/j.wneu.2019.01.016 30677572

[19] Kobata H Ikeda N. Recent updates in neurosurgical interventions for spontaneous intracerebral hemorrhage: minimally invasive surgery to improve surgical performance. Front Neurol 2021; 12: 703189. 34349724 10.3389/fneur.2021.703189 PMC8326326

[20] Patel NM Tran QK Capobianco P , et al. Triage of patients with intracerebral hemorrhage to comprehensive versus primary stroke centers. J Stroke Cerebrovasc Dis 2021; 30: 105672. 33730599 10.1016/j.jstrokecerebrovasdis.2021.105672

[21] Klaas JP Braksick S Mandrekar J , et al. Factors associated with the need for intensive care unit admission following supratentorial intracerebral hemorrhage: the triage ICH model. Neurocrit Care 2017; 27: 75–81. 28028788 10.1007/s12028-016-0346-7

[22] Cooley SR Zhao H Campbell BCV , et al. Mobile stroke units facilitate prehospital management of intracerebral hemorrhage. Stroke 2021; 52: 3163–3166. 34187178 10.1161/STROKEAHA.121.034592

[23] Mattila OS Ashton NJ Blennow K , et al. Ultra-early differential diagnosis of acute cerebral ischemia and hemorrhagic stroke by measuring the prehospital release rate of GFAP. Clin Chem 2021; 67: 1361–1372. 34383905 10.1093/clinchem/hvab128

[24] Forsberg S Höjer J Ludwigs U , et al. Metabolic vs structural coma in the ED – an observational study. Am J Emerg Med 2012; 30: 1986–1990. 22795990 10.1016/j.ajem.2012.04.032

[25] Kleindorfer DO Miller R Moomaw CJ , et al. Designing a message for public education regarding stroke: does FAST capture enough stroke? Stroke 2007; 38: 2864–2868. 17761926 10.1161/STROKEAHA.107.484329

[26] Oostema JA Chassee T Baer W , et al. Accuracy and implications of hemorrhagic stroke recognition by emergency medical services. Prehosp Emerg Care 2021; 25: 796–801. 33026277 10.1080/10903127.2020.1831669

[27] Keselman B Berglund A Ahmed N , et al. Analysis and modelling of mistriage in the Stockholm stroke triage system. Eur Stroke J 2022; 7: 126–133. 35647317 10.1177/23969873221077845 PMC9134772

[28] Carrera D Gorchs M Querol M , et al. Revalidation of the RACE scale after its regional implementation in Catalonia: a triage tool for large vessel occlusion. J Neurointerv Surg 2019; 11: 751–756. 30580284 10.1136/neurintsurg-2018-014519

[29] Turc G Bhogal P Fischer U , et al. European Stroke Organisation (ESO) – European Society for Minimally Invasive Neurological Therapy (ESMINT) guidelines on mechanical thrombectomy in acute ischaemic stroke endorsed by Stroke Alliance for Europe (SAFE). Eur Stroke J 2019; 4: 6–12. 31165090 10.1177/2396987319832140 PMC6533858

